# Carotid artery intima-media thickness and hypertensive heart disease: a short review

**DOI:** 10.1186/s40885-017-0063-3

**Published:** 2017-04-02

**Authors:** Costan G. Magnussen

**Affiliations:** 1 Menzies Institute for Medical Research, University of Tasmania, Private Bag 23, Hobart, 7001 Tasmania Australia; 2 Research Centre of Applied and Preventive Cardiovascular Medicine, University of Turku, Turku, Finland

**Keywords:** Hypertension, Carotid artery intima-media thickness, End organ damage, Blood pressure, Review

## Abstract

Sustained by its relative ease of assessment, carotid artery intima-media thickness (cIMT) has emerged as an important surrogate marker of target organ damage in hypertensive heart disease over the last three decades. However, the prognostic utility of cIMT in hypertensive heart disease differs depending on its application. This review outlines cIMT and its prognostic utility among patients with hypertensive heart disease. It provides an overview of limitations of cIMT and areas for future research.

## Background

Since the method was first proposed in the mid 1980s [1], the ultrasonic evaluation of the combined intimal and medial layers of the common carotid arteries have garnered substantial scientific and clinical support as an early, preclinical, vascular endpoint. Non-invasive, inexpensive, reproducible, and with prognostic utility among the asymptomatic and diseased, it is increasingly being used as a surrogate outcome or marker of target organ damage, or used as a tool to base treatment strategies. Although the use of carotid intima-media thickness (cIMT) is pervasive, it is not without controversy.

**Fig. 1:**
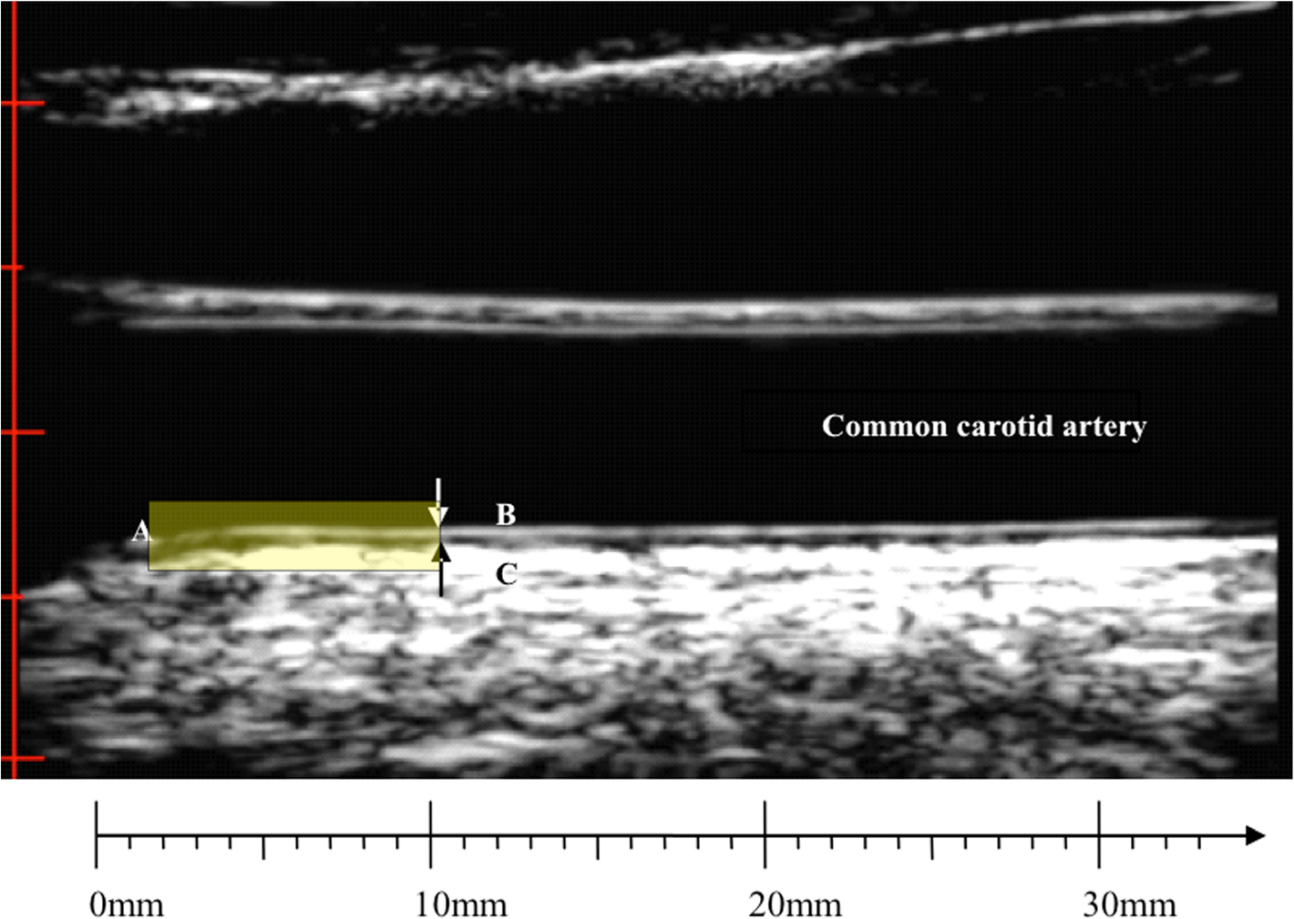
B-mode ultrasound image of the common carotid artery (CCA): **a** border of carotid bulb widening (0 mm), (**b**) CCA far wall lumen-intima interface, (**c**) media-adventitia interface. Definitions of the CCA segment for measurement differ depending on study and convention, but are typically measured in the vicinity 0–10 mm proximal to the border of the bulb widening (**a**) (measurement area highlighted by *yellow box*). CCA IMT is taken as the distance between (**b**) and (**c**)

Measured from B-mode ultrasound images as the distance between the intima-lumen interface and the media-adventitia interface (Fig. 1), there have been substantial methodological differences among studies in their approach to determining cIMT. These differences include the artery examined (left, right, or both), arterial segments to be examined (common carotid, internal carotid, bifurcation), and the position of the measurements within these segments, the phase of the cardiac cycle, the walls (near or far), whether plaque is included in the measurement, ultrasound technology, image angle, and the approach to measurement (mean, max, or mean of the max; average across all segments; type of analysis software – semi-automated vs. manual) [2]. Different approaches to cIMT acquisition and measurement have been shown to vary in terms of reproducibility, predictive utility, rate of change, susceptibility to drug treatment, and the ability to obtain images for measurement [3]. Recent progress toward standardising approaches to image acquisition and measurement of cIMT are proving useful [4–7] but more work is required to ensure these approaches are well integrated and appropriate for different research areas and questions.

In the hypertensive patient, identification of asymptomatic target organ damage may help inform treatment decisions, and refine or reclassify an individual’s cardiovascular disease risk. cIMT is one surrogate marker of target organ damage in the hypertensive patient that is considered in guideline statements by several authorities. The following will briefly overview the utility of cIMT for the hypertensive patient.

### Pathophysiology

Traditional ultrasound is unable to differentiate the intima and media as separate entities [8]. Therefore, an increased cIMT can be a result of a thicker intima, media, or both. Processes involved in intimal thickening are largely thought to mimic those in the pathogenesis and progression of atherosclerotic plaques, whereas hypertrophy of the media layer is primarily related to hypertension unrelated to atherosclerosis [7, 9]. Although thickening of the intima and media can occur by separate pathways, the elastic carotid artery has a relatively small media compared with muscular arteries and thus an increased cIMT is thought to largely denote intimal rather than medial thickening [10] and might explain the close association between cIMT and atherosclerotic-related cardiovascular events [11]. However, cIMT is considered a separate phenotype to atherosclerotic plaque formation and progression [7], with different clinical utility [12], and as such, should be measured in areas free of plaque [4].

### Prevalence

Prevalence data are not readily available because there is no widely accepted cut-off for what constitutes an adverse cIMT value. However, the European Society of Hypertension (ESH)/European Society of Cardiology (ESC) guidelines for the management of hypertension suggest a value greater than 0.9 mm as being a *conservative estimate* of asymptomatic organ damage [13]. Before criteria for what defines an abnormal cIMT can be established, there is need for measurement consensus and population reference values. In recent years there has been a concerted effort to standardise approaches to image acquisition and analysis with one example the initiation and routine update to the Mannheim Consensus statement [4]. Moreover, recent attempts to establish cIMT reference values among diseased and healthy populations have been notable, with the most comprehensive of these using data from approximately 25,000 individuals across 14 countries [14]. Interestingly, these data showed that a cIMT value of greater than 0.9 mm advocated by the ESH/ESC equates to values above the 90th percentile among the healthy, though very few less than 70 years of age would meet this criterion level. Because of the large age and sex effect on cIMT, suggestion of race or ethnicity differences in cIMT [15–18], as well as the variability introduced by different scanning and measurement protocols, any cut-points, if they were to be established, will likely need to recognise these differences.

### Clinical and prognostic significance

Since introduction of the technique, a plethora of data has emerged showing associations between traditional and emerging cardiovascular disease risk markers with cIMT and that cIMT associates with disease elsewhere in the vascular tree [19]. As with cardiovascular events, the association between systolic blood pressure and cIMT tends to be linear [20]. Irrespective of other risk factors, children with elevated blood pressure have higher adult cIMT than their counterparts [21], but those who are able to amend their elevated blood pressure status in the time between childhood and adulthood have a similar prevalence of high cIMT in adulthood as those that never had elevated blood pressure [22]. Clinical trials of antihypertensive medications have shown cIMT to regress or progress at a decreased rate among those receiving best therapy compared with those on different regimes or placebo [23–26].

There is substantial evidence that has demonstrated the utility of cIMT to predict incident vascular events in the asymptomatic [27] and diseased, including the hypertensive patient, with a 1-standard deviation increase in cIMT shown to associate with an 8% increased risk of myocardial infarction and 19% increased risk of stroke [28]. Nevertheless, the added value of cIMT measurements over and above risk factors included in the Framingham Risk Score, is inconsistent [29]. These inconsistencies, and the prevalent measurement issues, were the main weaknesses cited by the 2013 American College of Cardiology/American Heart Association guideline on the assessment of cardiovascular risk as reason not to measure cIMT [30]. Indeed, the ESH/ESC in their 2013 guidelines [31] were more cautious in their advice for cIMT measurement among hypertensive patients than in their 2007 release [13]. For hypertensive patients, the caution appears warranted following recent results from the IMPROVE (Immediate Management of the Patient with Ruptured Aneurysm: Open Versus Endovascular repair) cohort that showed area under the receiver operating curve, or c-statistic, to predict myocardial infarction and stroke was virtually unchanged when cIMT was added to the Framingham Risk Score [28]. Net reclassification improvement (NRI), which assesses the proportion of patients correctly reclassified to either a higher or lower risk, with the addition of cIMT was also low (1.4%). There has been suggestion that cIMT might benefit those of intermediate risk based on the Framingham Risk Score, and though IMPROVE found some statistical evidence of improved reclassification among hypertensive patients, the effect was again negligible (NRI = 5.6%).

### Future research questions/needs

Measurement standardisation and the establishment of population norms are needed, with strong recent progress being made in these areas. Progress is also being made with respect to the clinical utility of cIMT to reclassify risk among certain groups. Though the data suggest that cIMT, if it were to be used, might be most useful for reclassifying risk status amongst those of intermediate risk of a future event, data are not available as to whether the reclassification has a measurable impact on morbidity or mortality or whether the costs associated with scanning for cIMT outweigh the associated benefits, if indeed this is shown to be the case. Clinical studies should assess whether identifying an abnormal cIMT among the hypertensive patient asymptomatic of other target organ damage would improve treatment related outcomes. Particularly among the hypertensive patient, the utility of intima-media thickness measurements from other arterial sites such as in more muscular arteries that tend to have a thicker medial layer might provide additional prognostic utility over cIMT [11]. Interest has been gathering for the potential of cIMT to be used as a surrogate outcome for intervention trials, with a positive result acting as a trigger to initiate large-scale and costly testing of these interventions with the desired outcome of morbidity and mortality [32]. The potential use of cIMT in the paediatric setting to assess and monitor target organ damage in high-risk children has been reported [3, 33–35]. However, the application of cIMT to the paediatric setting has limitations that mimics those for adult populations – namely, lack of normative values and best measurement protocols, poor understanding of which cIMT segments provide best diagnostic utility, and its cost-effectiveness for routine monitoring.

## Conclusion

Measures of cIMT are elevated amongst those with hypertensive heart disease. Although individuals with a higher cIMT are at increased risk of clinical cardiovascular outcomes, the prognostic utility of cIMT differs by application and whether or not other risk factors are considered in the prediction. Further standardisation in measurement protocols, the subsequent establishment of a clear level of cIMT beyond which indicates target organ damage, and cost-effectiveness analysis of routine scanning of cIMT may provide additional information on the clinical usefulness of cIMT measurements for the hypertensive patient.

## Abbreviations

cIMT: Carotid artery intima-media thickness
ESC: European society of cardiology
ESH: European society of hypertension
IMPROVE: Immediate management of the patient with ruptured aneurysm: open versus endovascular repair
NRI: Net reclassification improvement
